# What Is the Added Value of Carotid CEUS in the Characterization of Atherosclerotic Plaque?

**DOI:** 10.3390/medicina60030375

**Published:** 2024-02-23

**Authors:** Andrejs Lioznovs, Maija Radzina, Laura Saule, Peteris Einars Grinbergs, Aigars Lacis

**Affiliations:** 1 Radiology Research Laboratory, Riga Stradins Universtity, LV-1007 Riga, Latvia; andrejs.lioznovs@rsu.lv (A.L.); laura.saule@rsu.lv (L.S.); peteris.grinbergs99@inbox.lv (P.E.G.); 2 Diagnostic Radiology Institute, Pauls Stradins Clinical University Hospital, LV-1002 Riga, Latvia; 3 Department of Vascular Surgery, Pauls Stradins Clinical University Hospital, LV-1002 Riga, Latvia; aigras.lacis@stradini.lv

**Keywords:** duplex Doppler ultrasonography, CEUS, unstable plaque, stenosis, neovascularization, atherosclerosis

## Abstract

*Background and Objectives*: Unstable atherosclerotic plaque in the arteries is one of the main risk factors for cerebral ischemia. Duplex ultrasound is a frequently used diagnostic method, but it has some limitations for microvascularization and neovascularization evaluation. The aim of this review was to evaluate the role of the new multiparametric US method—contrast-enhanced ultrasound (CEUS)—in atherosclerotic plaque instability verification. *Materials and Methods*: Original studies, reviews, and meta-analyses were included in this article. A total of 53 studies were retrieved; 29 were included in this study. *Results*: Carotid artery CEUS as a part of the multiparametric ultrasound method shows promising results and provides additional characteristics of soft- and high-risk atherosclerotic plaques; it can be advised in clinical practice for patients with carotid artery soft- and high-risk plaques. However, there are some limitations, such as extensive calcinosis with important acoustic shadows in carotid atherosclerotic plaque neovascularization diagnostics by CEUS. The added value of CEUS in the characterization of atherosclerotic plaque is that it indicates regions with high neovascularization and visualizes ulcerations on plaque surfaces, suggestive of increased instability risk.

## 1. Introduction

Carotid artery stenosis is one of the main risk factors of ischemic stroke, contributing to up to 10–20% of all strokes or transient ischemic attacks [1]. Several studies have shown that high plaque neovascularization is associated with a higher risk of symptomatic stenosis [2,3]. Few imaging techniques can be used to detect and quantify this neovascularization. With encouraging findings, the complementary ultrasonographic technique known as contrast-enhanced ultrasonography (CEUS) was recently launched for the assessment of carotid disease. As a result, official recommendations for its application were published [4]. Carotid artery plaque contrast-enhanced ultrasound is a medical imaging technique that uses ultrasound waves and a contrast agent to produce detailed images of the carotid artery and any plaque buildup within it. Plaque buildup within the carotid artery can lead to blockages and increase the risk of stroke, so it is important to accurately identify and assess the severity of any such buildup. 

The CEUS procedure involves injecting a contrast agent into the patient’s bloodstream, which helps to highlight the carotid artery and any plaque within it during the ultrasound exam. It leads to more accurate assessment of the size, location, and characteristics of the plaque buildup. CEUS is a non-invasive and relatively safe imaging technique, making it a useful tool for monitoring patients with known or suspected carotid artery disease. With the CEUS method, it is possible to differentiate between pre-occlusive stenosis and carotid occlusion and to grade a stenosis, and also it is possible to visualize the carotid system successfully. Additionally, CEUS can be utilized to highlight characteristics of carotid plaques that are vulnerable, such as intraplaque neovascularization and ulceration. CEUS can assess carotid dissection, inflammatory diseases, and surgical sequelae in addition to carotid atherosclerotic disease [5]. Feinstein first reported the feasibility of using contrast-enhanced ultrasound to identify intraplaque neovascularization (IPN) [6]. Recently, many studies have shown that the CEUS method can significantly improve the imaging effect of IPN. Dong et al. concluded that CEUS had high sensitivity and specificity in the diagnosis of atherosclerotic carotid plaque neovascularization [7]. A study by Huang et al. shows that there is a correlation between the grade of plaque enhancement and the risk of ischemic stroke. The data suggested that the presence of neovascularization is a marker for unstable plaque [8]. Overall, carotid artery plaque contrast-enhanced ultrasound can play an important role in the diagnosis and management of carotid artery disease, helping healthcare providers to make informed decisions about treatment and to reduce the risk of stroke.

## 2. Materials and Methods

In the present paper, a comprehensive literature search of PubMed, Google Scholar, and Scopus databases was conducted with the following key words: “carotid”, “artery”, “CEUS”, and “contrast-enhanced ultrasound”. The search strategy included analysis of papers with the mentioned key words. The results were subdivided into subjects and classified into “technical requirements”, “neovascularization detection”, “patient selection”, and “limitations” subdivisions; therefore, all articles which included the subdivision topics were included in this review. The search was updated from 2015 until November of 2023, and the references of the retrieved articles were explored. Original studies, reviews, and meta-analyses were included in this article. A total of 53 studies were retrieved; 29 were included in this study. To avoid bias, only studies with histological reference as the gold standard were included, and all of the MESH terms should have been present in the titles or abstracts.


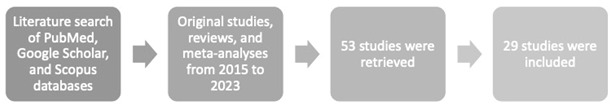


## 3. Results

### 3.1. Technical Requirements

All patients described in the studies underwent carotid artery plaque contrast-enhanced ultrasound. The following steps were needed for a successful CEUS procedure: first, the ultrasound device needed to be switched to contrast mode. The patients were in a supine position. Ultrasound examination was performed using a high-frequency linear probe and a non-linear pulse inversion technique. The frequency of linear probes in the studies varied from 2 to 14 MHz [9,10,11]. The contrast-enhanced ultrasound imaging application included a low mechanical index (from 0.06 to 0.21) to avoid early bubble destruction and harmonics with pulse inversion to optimize the depiction of the IV contrast agent and to minimize echoes from the surrounding tissues [12,13]. 

In most of the studies, the contrast suspension that was used was SonoVue (Bracca, Italy). In two studies, the agent used was Sonazoid^®^ (GE Healthcare, Oslo, Norway) [14,15] and, in one other study, perfluorocarbon-exposed sonicated dextrose albumin (PESDA) [16].

The volume of contrast media varied from 1 mL to 2.5 mL [17,18], and in most cases, it was diluted in 4–10 mL of saline [8,10]. In some studies, a double contrast media injection (2 mL and 4 mL) was performed [11]. In a study by Zhou et al., the time gap between the injections was approximately 3 min: an initial bolus injection was quickly performed, then the second injection was performed slowly and was followed by 5 mL of normal saline to flush out the contrast from the vein [17].

The probe was fan-shaped and gently oscillated during the examination to maximize the improvement of every area of the plaque. If further contrast was required to finish the observation, the previously described procedure was carried out. Following the injection of the contrast agent, the establishment of neovascularization within the plaque was indicated if dotted or short linear microbubble hyperechoes moved in a linear pattern around or inside the plaque. After injection, cine loops were recorded, starting from the time when the contrast agent could be observed in the carotid lumen. Following the infusion of the contrast agent, the lumen of the carotid artery was enhanced, resulting in visualization of enhanced plaque. The mean scanning time varied from 2 to 10 min [12,14,15]. In some cases, two videotapes were recorded for every injection: the early “dynamic” phase and the late “flash” phase, performed with six high mechanical index impulses [11].

All technical aspects of the contrast-enhanced ultrasound of the carotid artery in the included studies are described in Table 1.

### 3.2. Neovascularization Detection

The grading of neovascularization detection varies from study to study. Zhou et al. conducted a study where the presence of blood flow “activity” was identified on the basis of the dynamic movement of the echogenic reflectors (microspheres) in the intraplaque microvessels. Intraplaque neovascularization (contrast agent enhancement) was categorized using a modified grading scale and classified as class 1 (non-neovascularization) or class 2 (neovascularization) [17]. A similar two-grade neovascularization detection classification was used in a study by Zhu et al.: those with enhancement (grade 2) of carotid plaque and those without enhancement (grade 1) of carotid plaque [22]. In a study by Xiong et al., grade 1 was defined as no enhancement within the plaque or enhancement confined to the adventitial side of the plaque and/or the shoulder, and grade 2 was enhancement reaching the plaque core or extensive contrast enhancement throughout the plaque [21]. 

Qualitative analysis was used in a study by Fresili et al. to award scores on a scale of 1 to 3. The scores were as follows: score 1 was for no contrast enhancement; score 2 was for enhancement limited to the adventitial or peripheral region of the plaque; and score 3 was for diffuse intraplaque contrast enhancement. Carotid plaques are considered severe in terms of stenosis >70% and are vulnerable in cases of superficial ulceration and/or enhancement of secondary plaques or internal plaques (scores 2–3) [12]. A similar three-level rating system was also proposed in the study by Staub et al.; they defined grade 1 as having no microbubbles in the plaque or bubbles limited to the outer side; grade 2 presented moderate intraplaque enhancement with microbubbles migrating on the outer side of the plaque shoulder; and grade 3 exhibited extensive intraplaque enhancement with the clear appearance of microbubbles migrating toward the plaque core [3]. Amamoto et al. used a three-level grading system from 0 to 2 and defined grade 0 as having no visible dots or string-like microbubbles within the plaque; grade 1 was defined as having moderate microbubbles confined to the shoulder or adventitial side of the plaque; and grade 2 was determined by extensive microbubbles throughout the plaque [14]. In another study performed by Iezzi et al., grade 1 was defined as no enhancement within the plaque; grade 2 was enhancement confined to the adventitial side of the plaque and/or the shoulder; grade 3 was extensive flow of droplets of contrast media through the plaque core. In that study also, late-phase imaging was performed, and grade 1 meant enhancement while grade 2 meant no enhancement [11].

In some studies, neovascularization was classified in four grades. In a study by Schmidt et al., assessment of intraplaque neovascularization followed the classification of Huang and co-workers [19]: grade I—non-enhancement; grade II—arterial wall vasa vasorum enhancement; grade III—arterial wall vasa vasorum as well as plaque shoulder enhancement; and grade IV—extensive and internal plaque enhancement. For clinical simplicity, Schmidt et al. dichotomized CEUS findings into carotid stenosis with low (‘A’; grades I and II) and high (‘B’; grades III and IV) intraplaque contrast agent enhancement [19].

All grading of neovascularization detection in the included studies are described in Table 2.

### 3.3. Patient Selection

The criteria for including patients in carotid artery CEUS studies are different among the studies; in some studies, the subjects were patients with plaques detected by Doppler ultrasound; in some studies, they were symptomatic patients who were scheduled for carotid endarterectomy, but in some studies, patients with ischemic stroke were enrolled.

A study conducted by Iezzi et al. included 50 consecutive patients, both symptomatic and asymptomatic, who were referred to the department in order to receive a carotid endarterectomy (TEA) [11]. Huang et al. carried out a study in which 81 patients with ischemic stroke and 95 patients without stroke who had soft atherosclerotic plaques in the internal carotid artery were studied. That study shows that there is a correlation: the higher the grade of plaque enhancement, then the higher the risk of ischemic stroke. The data suggest that the presence of neovascularization is a marker for unstable plaque [8]. In a study by Xiong et al., 104 patients with carotid plaques were studied with standard and contrast material-enhanced ultrasonography (US). Among the 104 patients, 34% had transient ischemic attack and/or cerebrovascular ischemic stroke. In that study, plaque enhancement was found in 80% of symptomatic patients and in 30% of asymptomatic patients. The conclusion shows that symptomatic patients had more intense contrast agent enhancement in the plaque than asymptomatic patients, suggesting that contrast-enhanced carotid US may be used for plaque risk stratification [21]. 

Also, the criteria of inclusion and exclusion in the studies are variable. In another study represented by Huang S et al., 38 patients who were selected for carotid endarterectomy were enrolled in the study. The inclusion criteria included the following digital subtraction angiography examination findings suggestive of Benjamin et al.: moderate lumen stenosis (that means a stenosis rate of 50–69%) with stroke symptoms; moderate lumen stenosis (that means a stenosis rate of 50–69%) without any stroke symptoms, with imaging findings suggestive of vulnerable plaque; severe lumen stenosis (that means a stenosis rate of 70–99%); and incomplete occlusion. The exclusion criteria were the following: patients with non-atherosclerotic carotid stenosis (for example, aortitis and iatrogenic stenosis); patients with severe systemic diseases (for example, cardiac, hepatic, and renal insufficiency, malignant tumor, or hematologic disease); patients with incomplete specimen acquisition; and patients with the inability to sign the informed consent [9,26,27,28,29].

In some studies, the patient group was patients with recent ischemic events—ischemic stroke or transient ischemic attacks (TIA). In a study performed by Huang PT et al., 86 patients with ischemic stroke were recruited. Ischemic stroke was defined as focal neurological symptoms that lasted >24 h with or without persisting disabilities together with a computer tomography scan positive for ischemic lesions, in the absence of a cardiac source. For a patient to be included in the study, the stroke had to be recent, defined as not more than 30 days old. All patients had at least one soft plaque in the common carotid artery wall, its bifurcation, or the internal carotid artery, on the side relevant to the infarct. In that study also, a control group was recruited. Ninety-seven controls with soft carotid plaques were selected from 1556 patients referred for thyroid US examination at the same hospital. History of stroke or other cardiovascular disease was assessed by the investigators. Individuals reporting a positive history of stroke were not eligible, whereas those reporting a positive cardiovascular history other than stroke were eligible [8]. In a study by Li et al., the following TIA patients were enrolled: patients who had a past history of TIA. According to the American Stroke Association (ASA) guidelines (2009 version), TIA is defined as a brief episode of neurological dysfunction resulting from cerebral or retinal ischemia, with clinical symptoms typically lasting less than 1 h and without evidence of acute infarction on radiological imaging methods. Also, in that study, patients had carotid plaques that were determined by duplex ultrasound with plaque thickness ≥2.5 mm, and the patient age was >45 years [10]. 

In a study by Schmidt et al., 19 patients with high-grade carotid stenosis who were scheduled to undergo carotid endarterectomy (CEA) were enrolled in the study. Patients were classified according to the North American Symptomatic Endarterectomy Trial criteria (NASCET) as having asymptomatic or symptomatic ICA stenosis [30]. Symptomatic ICA stenosis is defined by the occurrence of neurologic symptoms that were referable to the ipsilateral carotid artery within the preceding 120 days. None of the patients suffering from asymptomatic ICA stenosis had suffered a previous ischemic event attributable to carotid stenosis [19].

### 3.4. Limitations

As with all methods, the CEUS method also has limitations. First of all, it is the ultrasound specialist/operator’s experience and skills that had a certain impact on the results of the ultrasound diagnosis. Subjective factors and operator experience had a great influence on the visual score of neovascularization in plaque. Also, there is a limitation due to patient appearance—limited penetration depth, which may be a challenge in obese patients [7,31,32].

Secondly, another limitation is related to the complicated process in which ultrasound waves and a contrast agent produce detailed images of the carotid artery and a plaque within it. A small number of microbubbles will be destroyed by the incoming ultrasound wave, and when this occurs, capillary damage may ensue. This in turn recruits vascular endothelial growth factor (VEGF)-producing inflammatory cells, stimulating neovascularization [33]. This phenomenon may be a deterrent to the use of CEUS in diagnostics due to the theoretical risk of potentiating plaque instability by promoting angiogenesis. However, at the low energies employed for CEUS, very few microbubbles will be affected, and this is therefore a theoretical risk [34].

One limitation of CEUS that Fresili et al. reported was its limited ability to evaluate the composition of plaques. More recently, intriguing new software has been released in the U.S. One example is 3D arterial analysis, which uses the composition of the plaque to determine the volume of carotid stenosis and identify the places that are vulnerable [12]. Another potential limitation was brought to light by Li et al. For patients who had several plaques, only the largest plaque was chosen after taking into account the contrast media dose limits and picture quality. This could include a few possible sources of bias in the research [10].

### 3.5. Results of Studies

The method accuracy of carotid artery CEUS is relatively high. In a study by Fresili et al., the sensitivity was 90.1%; the specificity was 96.7%; the PPV was 98.5%; and the NPV was 80.6%, with a total AUC of 93% [12]. Huang et al. came to the conclusion that contrast-enhanced ultrasound is a dependable and non-invasive examination method for assessing vulnerable plaques because it can show the amount of neovascularization within the plaque, indicate the risk of bleeding within the plaque, and determine the vulnerability of the plaque and because it has a high degree of agreement with pathological findings [9]. See Table 3 for all quantitative results of the included studies.

It was concluded that the presence of neovascularization is a marker for unstable plaque. A study by Huang S et al. shows that the higher the grade of plaque enhancement, the higher the risk of ischemic stroke [8]. Results of a study by Amamoto et al. showed that the intraplaque vessel size on CEUS was significantly associated with carotid plaque histology and may predict the process of plaque rupture and restoration [14]. 

A study by Xiong et al. indicated that symptomatic patients had more intense contrast agent enhancement in the plaque than asymptomatic patients, suggesting that contrast-enhanced carotid US may be used for plaque risk stratification [21]. Ventura et al. in their study concluded that, in making the differential diagnosis between occlusion and pseudo-occlusion of the cervical internal carotid artery, contrast-enhanced ultrasound with a second-generation contrast agent is significantly more effective than conventional Doppler ultrasound and is equally as effective as the gold standard (computed tomography angiography) [16].

Another important aspect is the unstable plaque’s role in patients with cardiovascular diseases. In a study by Mantella et al., it was concluded that carotid plaque neovascularization is a predictive factor of complex cardiovascular disease and possible future cardiovascular events. To sum up, the carotid IPN that was assessed with the CEUS method is a clinically useful tool for cardiovascular risk stratification in high-risk cardiac patients [35].

In several studies, CEUS is compared with other modalities. The advantages of CEUS include its limited costs, that it is a radiation-free method, and also its good availability. See Table 4, the comparison of CEUS with other methods [36].

## 4. Discussion

Duplex US with spectral analysis is the main modality for assessing carotid artery pathology due to its advantages, including diagnostic efficacy, affordability, repeatability, and accessibility. A recent technological advancement, contrast-enhanced ultrasonography, is a complementary ultrasonographic technology that offers certain advantages over the conventional technique. It has been used recently in the evaluation of carotid diseases [3]. 

The method accuracy of carotid artery CEUS is relatively high; it varies from 86% to 97% [11,16]. The indication for using carotid artery CEUS is width. 

CEUS as a method has some limitations:

- Limited Anatomical Information:

CEUS provides functional information about blood flow but may not offer as much anatomical detail as other imaging modalities. For a comprehensive evaluation of carotid artery anatomy, a combination of CEUS with other imaging techniques might be necessary.

- Operator Dependence:

The quality of CEUS images can be operator-dependent. Achieving optimal results requires skilled operators who are experienced in the technique. Inexperienced operators may struggle to obtain clear and accurate images of the carotid arteries.

- Artifact Interference:

Like any imaging modality, CEUS is susceptible to artifacts. Artifacts can arise from various sources, such as patient motion, respiratory movements, or the presence of calcifications. These artifacts can compromise the accuracy of carotid artery assessments.

- Limited Quantification:

While CEUS provides dynamic information about blood flow, it may have limitations in quantitative assessments compared to other imaging techniques. Quantifying parameters such as blood flow velocity or volume may be more challenging with CEUS.

- Contrast Agent Considerations:

The use of contrast agents in CEUS introduces the need to consider potential allergic reactions.

In the systematic review by Kopoyto et al., it was concluded that CEUS is a significant tool when evaluating the sonographic indicators of carotid plaque vulnerability, portraying surface irregularities, ulceration, intraplaque, neovascularization, and adventitial vasa vasorum development [36]. Accurate and timely detection of carotid atherosclerotic plaque and evaluation of its structural instability can help ensure timely optimal patient treatment algorithms, reduce stroke risk and incidence, and provide effective cost reduction, which are essential for surgical and conservative treatment decisions in individualized patient management.

## 5. Conclusions

CEUS as a part of the multiparametric ultrasound method shows promising results and provides additional characteristics of soft- and high-risk atherosclerotic plaques; it can be advised in clinical practice for patients with carotid artery soft- and high-risk plaques. Despite the excellent results described above, some limitation factors, such as extensive calcinosis with important acoustic shadows in carotid atherosclerotic plaque neovascularization diagnostics by CEUS, are still evident. CEUS remains an operator-dependent imaging technique, and bidimensional analysis of the plaque enhancement assumes that the longitudinal cross section of the plaque analyzed is representative of the whole carotid plaque. To sum up, the added value of CEUS in characterization of atherosclerotic plaque is that it indicates regions with high neovascularization and visualizes ulcerations on the plaque surface, suggestive of increased instability risk. 

## Figures and Tables

**Table 1:** Technical aspects of contrast-enhanced ultrasound of carotid artery.

Author	Year	Country	Patients (*n*)	Contrast Media	Probe Frequency	Mean Scanning Time	Mechanical Index
Iezzi et al. [11]	2015	Italy	50	Double contrast media injection (SonoVue (Bracco, Milan, Italy) 2 mL + 5 mL saline and after 10 min 4 mL + 5 mL saline)	8–14 MHz	6 min	0.09–1.3
Huang S et al. [9]	2021	China	38	1.5 mL contrast suspension (SonoVue, Bracca, Italy); flushed with 5 mL of saline	2–10 MHz	5 min	NM
Zhou et al. [17]	2013	China	46	5-mL solution—1 mL of the activated contrast agent (BR1; Bracco SpA, Milan, Italy; Definity, Lantheus Medical Imaging) and 4 mL of saline	2 MHz	NM	0.07
Huang PT et al. [8]	2010	China	176	SonoVue (Bracco, Milan, Italy) 2.4 mL + 10 mL saline	7 MHz	3 min	0.35
Fresili et al. [12]	2022	Italy	101	1.2 mL of SonoVue (Bracco, Milan, Italy), followed by a 10 mL saline flush	7.5 MHz	for at least 2 min	0.06–0.08
Schmidt et al. [19]	2017	Germany	19	2.4 mL SonoVue echocontrast agent (Bracco, Milan, Italy). The IV access was flushed with 5 mL 0.9% sodium chloride	5 MHz	NM	NM
Li et al. [10]	2019	China	112	A bolus of 2.5 mL of Sonovue + flushed with 5 mL of 0.9% sodium chloride	7–14 MHz	NM	0.2
Anamoto et al. [14]	2018	Japan	97	Sonazoid^®^	7 MHz	10 min (movie clips at 0; 1; 3; 5; 10 min)	NM
Cheung et al. [13]	2017	United Kingdom	24	SonoVue (Bracco, Milan) 1.2 mL/min	9 MHz	5–7 min	0.21
Jaipersad et al. [20]	2012	United Kingdom	10	SonoVue (Bracco, Milan, Italy) 6 mL	3–12 MHz	NM	0.08–0.1
Xiong et al. [21]	2009	China	104	SonoVue (Bracco, Geneva, Switzerland) 1.5 mL + 2–3 mL saline	6–8 MHz	5 min	0.13
Zhu et al. [22]	2013	China	312	SonoVue (Bracco, Geneva, Switzerland) 1.5 mL + 2–3 mL saline	6–8 MHZ	5 min	0.08–0.13
Hamada et al. [15]	2016	Japan	53	Sonazoid (perfluorobutane microbubbles; Daiichi Sankyo, Tokyo, Japan) 0.51 mg/kg	7 MHz	10 min (movie clips at 0; 1; 3; 5; 10 min)	NM
Ventura et al. [16]	2015	Brazil	72	Perfluorocarbon-exposed sonicated dextrose albumin (PESDA), 3 mL + 10 mL saline	7–12 MHz	NM	<0.4
Ning et al. [23]	2019	China	131	SonoVue (Bracco, Milan, Italy) 2.4 mL + 5 mL Saline	9–12 MHz	NM	0.05–0.08
Lyu et al. [18]	2020	China	51	SonoVue (Bracco, Milan, Italy) 2.5 mL + 5 mL Saline	NM	5 min	<0.1
Uchihara et al. [24]	2023	Japan	71	Sonazoid (0.01 mL/kg body weight) +10 mL Saline	4–9 MHz	5 min	0.2–0.3
Huang Z et al. [25]	2023	China	149	Sonazoid 1 mL + 5–10 mL Saline	6–8 MHz	5 min	0.24

NM—not mentioned.

**Table 2:** The grading of neovascularization detection.

Authors	Grading
Zhou et al., 2013 [17] Xiong et al., 2009 [21] Zhu et al., 2013 [22] Huang Z et al., 2023 [25]	Two levels: Grade 1 (non-neovascularization) Grade 2 (neovascularization)
Fresilli et al., 2022 [12] Amamoto et al., 2018 [14] Cheung et al., 2017 [13] Jaipersad et al., 2012 [20]	Three levels (from 0 to 2): Grade 0—no visible dots or string-like microbubbles within the plaque Grade 1— moderate microbubbles confined to the shoulder or adventitial side of the plaque Grade 2—extensive microbubbles throughout the plaque
Staub et al., 2011 [3] Iezzi et al., 2015 [11]	Three levels (from 1 to 3): Grade 1—no microbubbles within the plaque / bubbles included to the adventitial side Grade 2—moderate intraplaque enhancement with microbubbles at the adventitial side of plaque shoulder Grade 3—extensive intraplaque enhancement with the microbubbles moving to the plaque core
Ning et al., 2019 [23] Uchihara et al., 2023 [24]	Four levels (from 0 to 3): Grade 0—indicated no appearance of neovessel within the plaque Grade 1—revealed a limited appearance of neovessels within the plaque Grade 2—considered moderate neovessels within the plaque Grade 3—the presence of a pulsating, arterial vessel within the plaque
Huang S, 2021 [9] Li et al., 2019 [10] Huang PT et al., 2010 [8]	Four levels (from 1 to 4): Grade 1—no visible enhancement within the plaque Grade 2—enhancement at the base level of the plaque Grade 3—enhancement at the base and shoulder level of the plaque Grade 4—enhancement at the base, shoulder, and central region of the plaque

**Table 3:** Comparison of contrast-enhanced ultrasound of carotid artery studies results.

Author	Year	Country	Patients (*n*)	Sens	Spec	PPV	NPV	Acc	Conditions
Iezzi et al. [11]	2015	Italy	50	0.94	0.68	0.87	0.85	0.86	Both symptomatic and asymptomatic patients
Huang S et al. [9]	2021	China	38	0.89	0.80	0.93	0.73	0.87	Vulnerable plaques
Fresili et al. [12]	2022	Italy	101	0.90	0.97	0.98	0.81	0.94	Vulnerable plaques
Anamoto et al. [14]	2018	Japan	97	0.81	0.63	-	-	-	Intraplaque vessel size was analyzed
Xiong et al. [21]	2009	China	104	0.74	0.75	-	-	-	Both symptomatic and asymtomatic patients
Hamada et al. [15]	2016	Japan	53	0.91	0.69	0.68	0.92	-	Histological plaque rupture was analyzed
Ventura et al. [16]	2015	Brazil	72	1	0.9	0.97	1	0.97	Occlusion and pseudo-occlusion of carotid artery were analyzed
Huang PT et al. [8]	2010	China	176	0.82	0.8	-	-	-	Symptomatic patients with ischemic stroke
Ning et al. [23]	2019	China	131	0.82	0.77	-	-	-	Intraplaque neovascularization was analyzed
Lyu et al. [18]	2020	China	51	0.87	0.93	0.91	0.89	-	Intraplaque neovascularization was analyzed

Sens—sensitivity, Spec—specificity, PPV—positive predictive value, NPV—negative predictive value, Acc—accuracy.

**Table 4:** Comparison of CEUS with other imaging methods.

Imaging Techique	Application Scenarios	Advantages	Limitations
CEUS	Contrast agent indicates regions with high neovascularization Good visualization of plaque surface and ulceration	Non-invasive Radiation-free Good availability Limited cost	Operator dependency Variability Resolution
CT	High resolution allows for examination of plaque density and ulceration Most effective technique for detection of calcification	Non-invasive High resolution Reproducibility	Radiation Contrast agents Artifacts present due to calcification
MRI	Good differentiation between fibrous cap and necrotic lumen Intraplaque hemorrhage detection	Non-invasive Radiation-free High resolution Reproducibility	Gadolinium contrast is often needed Costs Time Availability

## Data Availability

Data are available from the corresponding author after appropriate review.
